# Immune checkpoint inhibitors for PD-1/PD-L1 axis in combination with other immunotherapies and targeted therapies for non-small cell lung cancer

**DOI:** 10.3389/fonc.2022.948405

**Published:** 2022-08-17

**Authors:** Ofek Mussafi, Jie Mei, Wenjun Mao, Yuan Wan

**Affiliations:** ^1^ Department of Cardiothoracic Surgery, The Affiliated Wuxi People’s Hospital of Nanjing Medical University, Wuxi, China; ^2^ The Pq Laboratory of BiomeDx/Rx, Department of Biomedical Engineering, Binghamton University-SUNY, Binghamton, NY, United States; ^3^ Department of Oncology, The Affiliated Wuxi People’s Hospital of Nanjing Medical University, Wuxi, China

**Keywords:** immunotherapy, PD-1, PD-L1, immune checkpoint inhibitor, lung cancer

## Abstract

It has been widely acknowledged that the use of immune checkpoint inhibitors (ICI) is an effective therapeutic treatment in many late-stage cancers. However, not all patients could benefit from ICI therapy. Several biomarkers, such as high expression of PD-L1, high mutational burden, and higher number of tumor infiltration lymphocytes have shown to predict clinical benefit from immune checkpoint therapies. One approach using ICI in combination with other immunotherapies and targeted therapies is now being investigated to enhance the efficacy of ICI alone. In this review, we summarized the use of other promising immunotherapies and targeted therapies in combination with ICI in treatment of lung cancers. The results from multiple animals and clinical trials were reviewed. We also briefly discussed the possible outlooks for future treatment.

## Introduction

One mechanism in which cancer weakens the body is through binding of the programmed death-ligand 1 (PD-L1) expressed on the surface of cancer cells with the programmed death 1 (PD-1) expressed on the surface of T cells. Over time this affinity leads to exhaustion of T cells and a weakened immune system through various signaling pathways due to the inhibitory nature of the PD-L1. So far, several mechanisms have been elucidated. PD-1/PD-L1 binding complex triggers the immunoreceptor tyrosine‐based switch motif (ITSM) found in the intracellular head of the PD-1 receptor to undergo phosphorylation. Next binding occurs between the high affinity T cell SHP2 molecule and the phosphorylated ITSM. This induces proximal T-cell receptor molecules such as zeta-chain (TCR)-associated protein kinase (ZAP70) to undergo dephosphorylation (1). ZAP70 binds directly with the major histocompatibility complex (MHC) receptor and plays a role in T-cell signaling. Dephosphorylation of ZAP70 leads to inhibition in downstream PI3K-AKT and RAS-ERK signaling, further reduces T-cell receptor (TCR)-mediated interleukin-2 (IL-2) and T cell proliferation, and thus promotes T-cell immunosuppression (2). Moreover, PD-1/PD-L1 interaction also leads to downregulation of LCK proto-oncogene (Lck) activity (3). Downregulation of Lck activity leads to a reduction of phosphorylated ZAP70 and ultimately to a downstream inhibition of the PI3K/Akt/mTOR pathway and the Ras/MEK/ERK pathway. Low Lck levels also leads to a reduction in phosphorylated CD3ζ, decreasing intracellular signaling and TCR surface expression. PKCθ is an enzyme found in the T cell that plays a role in intracellular signaling and is essential for T cell activation and IL-2 production. A reduction in Lck activity means less activated PKCθ availability and, therefore, a reduction in essential T-cell functions. Altogether, these pathways combine to exhaust T cells’ post PD-1/PD-L1 activation complex. Furthermore, PD-1 activation complex leads to downregulation of the CK2 molecule which phosphorylates the PTEN cytoplasmic domain. Higher levels of dephosphorylated PTEN lead to continued phosphatase activity and ultimately, inhibition of TCR activation signals. Together, these responses in T cells are linked to decrease function, proliferation, and overall, an exhausted phenotype.

PD-1/PD-L1 monoclonal antibodies (mAbs) trigger an antitumor response by blocking this binding complex between T cells and tumor cells. These mAbs bind directly to the PD-1 on T-cell membranes or the PD-L1 on tumor-cell membranes. Once binding occurs, these mAbs will stop the inhibition of the T-cell immune surveillance response. Moreover, they can increase the production of interferon-gamma (IFN-γ), IL-2, and interleukin-7 (IL-7) (4). IFN-γ is a cytokine found in T cells that plays a role in inducing and modulating several immune responses. IL-2 is another cytokine that has been shown to be induced *via* three different signaling pathways, i.e., JAK-STAT, PI3K/Akt/mTOR, and MAPK/ERK pathways, all of which are suppressed by PD-1/PD-L1 binding (5). IL-2 plays a role in T-cell immune regulation by converting T cells into Treg cells to prevent strong autoimmune response and enhance activation-induced cell death (AICD). It is also involved in increasing T-cell differentiation into effector T cells and memory B cells to fight off pathogens and tumor-associated antigens. IL-7 is an important cytokine involved in the development and growth of B cells and T cells. During early T-cell development, IL-7 plays a role as an important cofactor for V(D)J rearrangement of the T-cell receptor beta (TCRB) (6). Through the inhibition of the PD-1/PD-L1 binding complex and the up-regulation of these cytokines patients can achieve profound survival benefits including higher overall survival (OS) and objective response rate (ORR). Briefly, mAbs targeting the PD-1/PD-L1 axis has shown tremendous benefit in clinical trials and have been approved as second-line or first-line therapies for an increasing number of carcinomas, including lung cancer, melanoma, lymphoma, among others (7). Currently, FDA-approved drugs for use in PD-1 blockade include pembrolizumab, nivolumab, avelumab, and in PD-L1 blockade include atezolizumab and durvalumab. These drugs fall into a class of therapeutics known as checkpoint inhibitor blockade (ICB). ICB fall into a bigger class known as immunotherapies, which are considered one of the most important advancements in cancer treatment.

Since FDA approval of nivolumab and pembrolizumab, two mAbs targeting PD-1, for the treatment of advanced melanoma in 2014, countless studies on PD-1/PD-L1 have flooded the scientific community (8). This review will focus on lung cancers, specifically non-small cell lung cancer (NSCLC). It has been revealed that only 30% of NSCLC patients are diagnosed at stage I, for which the 5-year survival rate is 65%. In contrast, the 5-year survival rate for stage IV is only 5% (9).. Around 85% of all lung cancer cases are NSCLC (10). PD-1/PD-L1 drugs have shown promising benefits and low treatment-related adverse events (AE) for NSCLC in many clinical trials. A study of 495 NSCLC patients treated with pembrolizumab monotherapy achieved an ORR of 19.4%, and a median duration of response of 12.5 months, leading to pembrolizumab was approved as a single agent for the first-line treatment for NSCLC patient with PD-L1 TPS ≥1% and without driver gene mutations (11). Another randomized NSCLC study testing nivolumab in comparison to docetaxel demonstrated that progression-free survival (PFS), OS, and ORR were considerably improved with nivolumab irrespective of PD-L1 expression (12). Based on the findings, nivolumab was approved as second-line treatment of non-squamous advanced NSCLC after failure of prior platinum-based chemotherapy. When the PD-L1 inhibitor durvalumab was given to NSCLC patients after concurrent chemoradiotherapy, investigators saw an increased OS rate (66.3% in the durvalumab group *vs*. 55.6% in the control group), increased PFS (17.2 months for durvalumab group *vs*. 5.6 months in the control group), and increased median time to death or distant metastasis (28.3 months in the durvalumab group *vs*. 16.2 months in the control group) (13). Based on the findings, FDA approved durvalumab as the first maintenance therapy for stage III unresectable NSCLC.

Despite promising clinical benefits, a large percentage (>50%) of cancer patients still do not respond to the ICB. Mainly, the reasons for this can be attributed to a decreased number of tumor-infiltrating lymphocytes (TILs) and a lack of highly expressed PD-1/PD-L1 axis on all cancer cells. Therefore, colleagues speculate that patients with PD-L1 overexpressing tumor may have optimal treatment outcomes (14–16). Another major concern in ICB is the toxic AE, which are commonly experienced alongside a new growing list of immune-related adverse effects. In some instances, these AEs prove more harm than benefits, and, thus, the treatment must be discontinued. With these challenges being the major setbacks in ICB, modern therapeutic approaches are now looking to expand upon PD-1/PD-L1 inhibition in ways to minimize adverse events, while increasing treatment efficacy. Accordingly, researchers have been exploring combination strategies with other types of cancer therapy, such as chemotherapy, targeted therapy, radiotherapy, and immunotherapy (17, 18). Unlike chemotherapy and radiotherapy that damage tumors indiscriminately, immunotherapy and targeted therapy use specific protein-targeted approaches that reduce cytotoxicity to normal cells. This allows for lower rate of AEs in comparison. For instance, one study compared immunotherapy with the combination of immunotherapy and chemotherapy. The investigators found the combinational therapy demonstrated higher ORR (15.2% *vs*. 43.5%) and significantly longer PFS (4.6 *vs*. 15.5 months) in NSCLC patients (19). In another study, in comparison with chemotherapy alone, combinational therapy demonstrated a higher ORR (19.9% and 48.3%) and longer 2-year PFS (3.4% *vs*. 22%) in advanced NSCLC (20). Given immunotherapy is very promising, we particularly reviewed ICB in combination with other immunotherapy and targeted therapies in NSCLC, which includes cancer vaccine, mAb, oncolytic viruses, protein targeted compounds, immunomodulators, and adoptive cell transfer therapy. Going forward, we acknowledge that the scope of this research is vast with far too many significant treatments to cover in one review. Therefore, we dedicated our focus to combinations of immunotherapy with high potential for lung cancers.

## Combination with cancer vaccine

### Neoantigen-based vaccines

Modern vaccinations typically work by introducing a foreign substance or antigen in small or weakened doses into the body, which allows the immune system to develop antibodies specifically programmed to eliminate the antigen. Vaccines are now also being tested in cancer therapy. In cancer treatment, vaccinations use the antigens found in tumor cell membranes as therapeutic targets, which can be recognized and targeted by immune cells, thus triggering specific immune response against tumor cells (21, 22). Neoantigen vaccines work by identifying and targeting antigens found exclusively on the surface of cancer cells known as tumor-specific antigens (TSA). Every patient has a unique set of TSA, which requires personalized treatment typically targeting up to 20 unique neoantigens. Neoantigen-based vaccines promote neoantigen-specific CD4+ T cells and CD8+ T cells against neoantigen expressing cells. Subsequently, T cells can search, recognize, and kill cells harboring these antigens, achieving a powerful and specific anti-tumor response. This boost in the immune system in combination with ICB can potentially increase treatment efficacy. Indeed, T cell targeting neoantigens has been associated with anti-tumor activity and has long been believed to be effective targets for anti-tumor response (23, 24). In a recent study, neoantigens were successfully identified as targets through RNA sequencing of NSCLC tumor and blood samples harvested from patients. The RNA sequencing data were analyzed to identify mutations, genetic expression information, and human leukocyte antigen (HLA) typing in order for several mutated neoantigens characterized by strong HLA affinity to be chosen and tested (25). Through *in vitro* experiments and neoantigen reactive T cells (NRT)-induced cytotoxicity *in vivo* evaluation, they demonstrated NRT had responses against neoantigens with high HLA affinity. In mice models bearing the NSCLC tumors, they were able to show that targeted therapy against ACAD8-T105I, BCAR1-G23V, and PLCG1-M245L led to improved immune cell response, demonstrating the feasibility of treatment *in vivo*. In a stage III/IV NSCLC study with 24 patients, neoantigens-based personalized vaccination was developed based on predictions using a panel of 508 tumor-associated genes from tumor biopsies, with peptides also demonstrating high affinity to HLA class I and II, determined through HLA typing (26). Researchers were able to demonstrate OS and median PFS of 8.9 and 6 months, respectively. Five patients demonstrated vaccine induced CD8+ T cell responses against epidermal growth factor receptor (EGFR) neoantigen peptides, showing that personalized neoantigen vaccination is a feasible and safe method to increase immune response against tumor cells harboring EGFR mutations. Another study tested the efficacy of PD-1 inhibitor nivolumab in combination with personal neoantigen vaccine, NEO-PV-01 (27). This was the first time ICI was tested in combination with neoantigen vaccine in NSCLC patients. It was determined that the approach provided minimal toxic AEs, while specifically activating CD4+ and CD8+, MHC class I, and MHC class II against restricted neoantigen epitopes. It is noteworthy that only three patients with NSCLC were treated, and, therefore, further clinical data are needed to confirm the findings of this study and to demonstrate the feasibility of this combination approach.

### CIMAvax

EGFR and epidermal growth factor (EGF) play critical roles in healthy cell tissue development and homeostasis (28). EGFR falls into a receptor class that is heavily involved in a multimodal signaling cascade responsible for cellular migration, differentiation, and proliferation (29). Overexpression of EGFR occurs in ~60% of NSCLC patients and is associated with poor differentiation, increased tumor proliferation, higher incidence of metastases, and lower efficacy (30, 31). The EGFR/EGF complex has long been viewed as therapeutic targets in NSCLC. The mode of action behind these treatments involves inhibition of EGFR, cutting off tumors’ main mechanism to proliferate and undergo angiogenesis. Another form of treatment involves binding to the EGF directly to create a conformational change, decreasing the amount of available EGF in the bloodstream for cancer cell binding. CIMAvax-EGF vaccine is a recombinant Neisseria Meningitis B bacterium-derived outer membrane protein P64K, conjugated onto human recombinant EGF and using Montanide ISA51 as an adjuvant, leads to an upregulation of anti-EGF antibodies (32). Ultimately, the decrease in EGF in the bloodstream starves cancer cells, directly deregulating critical pathways involved in tumor growth, signaling, and differentiation. In phase III randomized study testing CIMAvax-EGF in advanced NSCLC patients that were previously treated with frontline chemotherapy, results showed that median OS in vaccinated and nonvaccinated patients was 12.43 and 9.43 months. Moreover, long-term survival in vaccinated and nonvaccinated after 2 years was 37% and 20%, and after 5 years was 23% and 0%, respectively (33). The researchers hypothesized that high level of EGF (>870 pg/ml) could be used as a predictive biomarker of CIMAvax efficacy. Interim results from a phase I/II study using nivolumab in combination with CIMAvax in advanced NSCLC showed ORR 44% (four out of nine) of patients with no AE of 3+, except in one patient due to nivolumab alone (34). Compared to nivolumab monotherapy, where ORR was reported as 23% (12 out 52). These findings indicate an improvement in efficacy for this combinational approach (35). Importantly, three out of the four patients had PD-L1 expression <1%, demonstrating success in cancer patients that normally exhibit poor prognosis with anti-PD-L1 treatments. It was determined that four doses of the GIMAvax-EGF vaccine were optimal, and the only dosing scheme where >50% of patients achieved sufficient antibody responders. Results from nivolumab/pembrolizumab in combination therapy with CIMAvax are awaited as the trial is still recruiting at the time of this publication (NCT02955290). Despite promising results, it is important to acknowledge that there exists a lack of data regarding the possibility of vaccine neutralization from the patient’s immune system, which should be further explored. Additionally, it would be interesting to see how anti-EGFR antibodies compared with CIMAvax-EGF.

### TG4010

MU1 is an antigen from a family of mucin. This glycoprotein plays a role in keeping pathogens out of the body through binding oligosaccharides to its extracellular domain (36). Overexpression of MU1 has been associated with lung, colon, breast, and pancreatic cancers (37). Additionally, biochemical differences exist between healthy MU1 expression and tumor associated MU1 expression. For example, MU1 transcriptional genes, such as STAT3, NF-kB, p53, and β-catenin are associated with tumor invasion, proliferation, and angiogenesis (38). TG4010 vaccine is a modified virus designed to express coding genes for MU1 and IL-2. This vaccine therapy deregulates critical pathways for NSCLC cell survival and, therefore, could prove effective in combination with ICB. In a phase II/III randomized, double-blind study testing stage IV NSCLC patients lacking an EGFR mutation while also expressing ≥ 50% of MUC1 on tumor cell surface, 222 patients received standard first-line chemotherapy in combination and without combination of TG4010 vaccine (39). Results showed that median PFS was 5.1 months in the group without TG4010 and 5.9 months in the group with TG4010. The adverse event was 4% in the combinational therapy group and 10% in the control group. Following up on the promising findings, two ongoing clinical trials are testing the feasibility of nivolumab in combination with TG4010 (NCT02823990) and triple arm nivolumab in combination with TG4010 and chemotherapy II with PD-L1 <50% (NCT03353675).

### Cell-based vaccines

Antigen-presenting cells (APC) such as mature dendritic cells (mDC) are now being utilized in cancer vaccine therapies. Normally, APCs function as a surveillance system in the body continuously monitoring the extracellular environment for antigens. Once an antigen is identified, they return to the lymph nodes and bind to T cells through MHC I and MHC II, which cause activation against the antigen. In cancer therapy, one approach requires the use of neoantigens or TSA found in the patients to transfect mDC *in vitro* creating APCs that promote targeting to specific neoantigens or TSA. Subsequently, once educated mDCs are transplanted into the patient, these mDCs can simulate CD4+ and CD8+ T cells against tumor cells. However, this approach has proven to be impractical and time-consuming. A new approach is using the patient’s intratumor as the neoantigen and creating vaccine response *in vivo*. This approach creates antitumor-specific CD8+ T cells by acting more of a primer releasing pro-inflammatory chemokines including CCL4, CCL5, CXCL10, and cytokines at the time of intra-tumoral administration, rather than an antigen-presenting platform helping induce the recruitment of immune cells, including T cells to the injection site (40, 41). T-cell activation can potentially demonstrate a key agonist that can help improve PD-1/PD-L1 treatments. Current ongoing clinical trials of combination mDC vaccine and PD-1/PD-L1 inhibitors include pembrolizumab in NSCLC (NCT03546361), pembrolizumab in solid tumors (NCT03735290), and Pembrolizumab in plasmacytoid dendritic cells (PDC) derived from NY-ESO-1 antigens (NCT03970746). The ongoing clinical trials testing vaccine-based therapeutics with ICI were listed in **Table 1**.

**Table 1:** Ongoing clinical trials testing vaccine-based therapeutics with PD-1/PD-L1 checkpoint inhibitors.

Clinical Trial	Cancer Type	Setting	Phase	VaccineAgent	Anti-PD-1/PD-L1	Primary Outcomes	N. Patients
NCT03639714	Advanced Solid Tumors	Personalize Neoantigen cancer vaccine	I/II	GRT-C901/GRT-R902	nivolumab/ipilimumab	AE, SAE, DLT, ORR, RP2D	214
NCT04266730	Squamous NSCLC	Personalized and Adaptive Neoantigen, Dose-Adjusted vaccine	I	PANDA-VAC	pembrolizumab	AE	6
NCT03953235	NSCLC	Personalized Vaccine targeting shared Neoantigens	I/II	GRT-C903/GRT-R904	nivolumab/ipilimumab	AE, SAE, DLT, ORR, RP2D	144
NCT04998474	NSCLC	Personalized Vaccine	II	FRAME-001	pembrolizumab	Antigen-specific immune response	15
NCT03380871	Advanced or Metastatic non-squamous NSCLC.	Neo-PV-01 plus pembrolizumab Plus Chemotherapy	I	NEO-PV-01	pembrolizumab	AE	38
NCT02897765	NSCLC	NEO-PV-01 + Adjuvant With nivolumab	I	NEO-PV-01	nivolumab	SAE, AE,	34
NCT02955290	NSCLC, Metastatic and Unresectable NSCLC	CIMAvax + pembrolizumab/nivolumab	I/II	CIMAvax	pembrolizumab, nivolumab	DLT, AE, OS	42
NCT02823990	NSCLC	TG4010 + nivolumab	II	TG4010	nivolumab	ORR	13
NCT03353675	NSCLC	TG4010 + chemotherapy + nivolumab	II	TG4010	nivolumab	ORR	39
NCT03970746	NSCLC	PDC*Lung01 + injectable pembrolizumab/pemetrexed	I/II	PDC*lung01	pembrolizumab	DLT	64
NCT03546361	NSCLC	Autologous Dendritic Cell-Adenovirus CCL21 Vaccine + pembrolizumab	I	CCL21 Vaccine	pembrolizumab	MTD/MAD, ORR	24
NCT03735290	NSCLC	ilixadencel + pembrolizumab	I/II	ilixadencel	pembrolizumab	AE, SAE, DLT, ORR	150
NCT03847519	NSCLC	ADXS-503 + pembrolizumab	I/II	ADXS-503	pembrolizumab	AE, DLT, anti-tumor activity	74

## Combination with mAb

### Anti-EGFR antibody

Overexpression of EGFR is commonly found in a variety of cancers including NSCLC. Gene mutations at the EGFR are responsible for continuous autophosphorylation and a continuous activated state, which, ultimately, leads to carcinogenesis. Currently, there are two common approaches by which targeted treatment to this complex occurs. One is through an anti-EGFR mAb binding directly to the extracellular domain of EGFR, which will be primarily covered here. The other is *via* small-molecule EGFR tyrosine kinase inhibitors (TKI), binding competitively with adenosine 5′ triphosphate to the intracellular catalytic head of EGFR, covered more in depth in the next section (42). The binding of both therapeutics downregulates continuous phosphorylation and activation pathways involved in oncogenic mutations. Additionally, one anti-EGFR mAb known as cetuximab suggests that inhibition of this receptor-ligand axis also leads to immune stimulation and is associated with counterregulatory mechanisms. Cetuximab is also thought to be linked in immune suppression feedback loops that include an increase in the expression level of immune checkpoint receptors like PD-L1 in cancer cells (43). These results suggest an additive effect that is ideal for increasing treatment efficacy for ICB by increasing levels of PD-1/PD-L1. A stage IV NSCLC phase Ib dose-escalating trial of necitumumab, an anti-EGFR mAb, combined with pembrolizumab demonstrated ORR of 23.4%, PFS of 4.1 months regardless of PD-L1 status (44). The OS of patients at 6 months was 74.7%. It is noteworthy that patients with PD-L1 expression of ≥1% had improved ORR and medium PFS when compared to PD-L1 negative patients. It was concluded that the approach was tolerable with no dose-limiting toxicities reported and provided better efficacy than treatment in monotherapy, particularly in tumors of <50% PD-L1 expression. In a phase II trial for NSCLC testing avelumab in combination with cetuximab and chemotherapy results from 43 patients demonstrated ORR of 30.2%, OS of 10 months, and medium PFS of 6.1 months (45). No significant toxic AEs occurred compared either Avelumab or Cetuximab alone. However, anti-EGFR mAb and anti-PD-1/PD-L1 combination approach is still in the early stage of testing, and, therefore, due to lack of data, these findings are hard to validate.

### Anti-VEGF/VEGFR mAb

Vascular endothelial growth factor (VEGF) is a protein that promotes angiogenesis. In the cases of malignant cells, VEGF/VEGFR is commonly overexpressed leading to rapid proliferation and expansion of tumor tissue. Inhibition of this receptor-protein complex acts as an angiogenesis antagonist, leading to a decrease in cancer metastases and proliferation. Results from Bevacizumab, an anti-VEGFA mAb, in combination with atezolizumab demonstrated ORR of 64% and medium duration of response of 10.4 months as a first-line chemo-free therapy in NSCLC with PD-L1 expression >50% (46). However, despite promising results, prolonged exposure to VEGF/VEGFR mAb can lead to adaptive resistance by tumors and create a path for new expansion mechanisms. Ongoing clinical trials with anti-VEGF/VEGFR mAb are summarized in **Table 2** with more results awaited to provide relevant information on the combination approach.

**Table 2:** Ongoing clinical trials testing VEGF targeted antibodies in combination with PD-1/PD-L1 checkpoint inhibitors.

Clinical Trial	Cancer Type	Intervention	Phase	VEGFAgent	Anti-PD-1/PD-L1	Primary Outcomes	N. Patients
NCT03786692	Stage IV NSCLC	carboplatin, + pemetrexed, + bevacizumab, +/- atezolizumab	II	bevacizumab	atezolizumab	PFS	117
NCT04124731	Advanced NSCLC	sintilimab plus anlotinib	II	anlotinib	sintilimab	ORR	98
NCT04471428	Metastatic NSCLC	cabozantinib + atezolizumab	III	cabozantinib	atezolizumab	OS	366
NCT03386929	Metastatic/Stage III NSCLC	avelumab + axitinib + palbociclib	I/II	axitinib	avelumab	DLT, AE, RR, DR, PFS, OS, SIM	130
NCT05078931	NSCLC	pembrolizumab + lenvatinib	II	lenvatinib	pembrolizumab	PFS	35
NCT04147351	NSCLC Stage III-IV	atezolizumab; bevacizumab	II	bevacizumab	atezolizumab	ORR	22
NCT04459663	NSCLC	toripalimab injection combined with axitinib	II	axitinib	toripalimab	Antitumor activity, ORR	50
NCT03971474	Recurrent NSCLC	ramucirumab + pembrolizumab	II	ramucirumab	pembrolizumab	OS	166

## Combination with targeted therapies

### Targeting tyrosine kinase

In addition to anti-EGFR mAb, a different approach exists targeting the EGF/EGFR complex, using TKIs instead. Activation of the oncogenic EGFR pathway in preclinical studies has shown enhancements in the susceptibility of lung tumors to anti PD-1 inhibitors in mice models, suggesting that EGRF TKIs in combination ICI may be a promising therapeutic approach, especially in EGFR mutated NSCLC (47). However, clinical trial data have been inconclusive with some reports finding AEs outweighing efficacy. In an EGFR mutation-positive NSCLC clinical trial, nivolumab in combination with Erlotinib (EGFR TKI) resulted in tolerable safety profile with an ORR of 19%, and durable response was observed in four out of 20 patients (48). A previously untreated stage IIIb/IV EGFR-mutant NSCLC study combining pembrolizumab and erlotinib reached an ORR rate of 41.7% with similar toxicities as pembrolizumab monotherapy (49). When comparing to a pembrolizumab monotherapy, where ORR was reported as 44.8%, no improvement in ORR was present for pembrolizumab alone (50). However, when compared to erlotinib monotherapy, which showed ORR of 22.7%, results do show a significant increase in ORR (51). This suggests that the combination approach has an increase in ORR when compared to EGF inhibitor alone, but no noticeable increase when compared to pembrolizumab alone. Importantly, different EGFR mutations can lead to different treatment outcomes. EGFR exon 18 G719, exon 19 K757R, exon 20 S768I, exon 21 G836S, and E746G mutations have been correlated to successful treatment outcomes in NSCLC patients, whereas tumors exhibiting exon 18 S720I mutation demonstrated poor clinical outcomes to erlotinib (52). It’s worth mentioning most anti-EGFR treated patients build an adaptive resistance to treatment, raising speculation on the viability of this combination approach. Nonetheless, further data are awaited with **Table 3** capturing the ongoing clinical trials of combination ICI with anti-EGF/EGFR therapeutics. In addition, anlotinib is a small molecule that acts as a receptor tyrosine kinase (RTK) inhibitor with multi-RTKs inhibition pathways including VEGFR-2 and VEGFR-3, fibroblast growth factor receptor (FGFR), stem cell-factor receptor (c-Kit), and platelet-derived growth factor receptor (PDGFR) (53). This agent may both inhibit angiogenesis and downregulate tumor expansion. Clinical trials have indicated that anlotinib significantly prolonged OS (9.3 *vs*. 6.3 months) and PFS (4.8 *vs*. 1.2 months) of patients with advanced NSCLC (54). Evidence shows that a combination approach can reverse PD-1/PD-L1 resistance when combined with nivolumab. In a phase Ib study of sintilimab in combination with anlotinib as first-line therapy, out of the 22 NSCLC patients enrolled in the study, 16 demonstrated an ORR of 72.7% and the 12-month PFS was 71.4% (55).

**Table 3:** Ongoing clinical trials testing EGF/EGFR TKI and targeted antibodies in combination with PD-1/PD-L1 checkpoint inhibitors.

Clinical Trial	Cancer Type	Intervention	Phase	EGF/EGFRAgent	Anti-PD-1/PD-L1	Primary Outcomes	N. Patients
NCT02924233	NSCLC	Sym004 + nivolumab	I/II	Sym004	nivolumab	DLT, RP2D	0
NCT04042701	NSCLC	DS-8201a + pembrolizumab	I	DS-8201a	pembrolizumab	DLT, ORR	115
NCT04976647	Squamous NSCLC	HLX07 + HLX10	II	HLX07	HLX10	ORR, PFS	156
NCT04646330	NSCLC	AK104+anlotinib	I/II	anlotinib	AK104	ORR	120
NCT02013219	NSCLC	erlotinib + atezolizumab	I	erlotinib	atezolizumab	DLT, RP2D,	52
NCT02947386	Recurrent NSCLC, Stage III-IV NSCLC	nimotuzumab + nivolumab	I/II	nimotuzumab	nnivolumab	DLT, ORR	48

### Targeting KRAS

KRAS mutant genes, the most frequent altered gene in NSCLC for years has been sought after as a therapeutic target with little success partly due to its high affinity for GTP and complex down streaming pathways (56). Recent FDA approval of KRAS G12C inhibitor, sotorasib has opened light on this treatment approach. Current dose exploration and dose expansion clinical trial combining sotorasib with pembrolizumab (NCT04185883) is undergoing, which included participants with KRAS p.G12C advanced non-small cell lung cancer. Upon completing the dose exploration, dose expansion may also proceed consisting of participants with KRAS p.G12C mutant advanced NSCLC. In addition, another trial on sotorasib in combination with pembrolizumab (NCT03785249) is also undergoing, which is a phase 1 evaluation of the safety, tolerability, and clinical activity in patients with KRAS G12C mutant unresectable or metastatic NSCLC.

### Targeting IDO1

Indoleamine 2,3-Dioxygenase 1 (IDO1) is an enzyme expressed by the IDO1 gene, which is responsible for catalyzing tryptophan into kynurenine *via* the tryptophan-kynurenine-aryl hydrocarbon receptor (Trp-Kyn-AhR) pathway. Tryptophan is associated with healthy T-cell function (57). Depletion of tryptophan inhibits mTORC1 signaling pathway, which leads to T-cell autophagy and the release of GCN2-mediated phosphorylation of eIF-2. Finally, the ripple effects induce cell-cycle arrest and death in T cells (58). Therefore, upregulation of the IDO1 gene is associated with increased immunosuppression due to T-cell apoptosis and increased metabolites of IDO1 in the tumor microenvironment (TME) (59). Furthermore, IDO1 overexpression has been observed after ICB in NSCLC patients, suggesting a possible role in the process of acquired resistance and has been hypothesized to negatively affect post-treatment prognosis (60). Clinical studies indicated that IDO1 inhibition combined with ICB may have added antitumor effects and heightened immune response. In a phase I/II trial, IDO-1 inhibitor epacadostat, in combination with pembrolizumab for pretreated advanced NSCLC, demonstrated ORR of 35% with generally good tolerability. Signs of toxicity were present as 11% of patients had to discontinue treatment due to AEs (61). However, a phase III trial of the same treatment demonstrated no advantage from the combination approach in comparison with pembrolizumab alone (median PFS of 4.7 *vs*. 4.9 months; ORR of 34% *vs*. 32%) (62). In a phase I study to explore the combination of navoximod (another IDO1 inhibitor) with atezolizumab for the first time as treatment for patients with advanced cancer, navoximod combined with atezolizumab demonstrated good tolerability and acceptable safety (63). In the dose escalation stage, six of 66 (9%) patients achieved PR, and 11 (17%) patients achieved SD, and the rate of treatment-related Grade ≥ 3 adverse event increases with increasing doses of navoximod (63). However, there was no clear evidence of benefit from the combination approach in comparison with atezolizumab alone. The mixed results from these trials indicated that IDO1 inhibition alone might not sufficiently induce T-cell activation. More clinical trials are underway to better understand IDO1 inhibition and its potential in combination with ICI (**Table 4**).

**Table 4:** Ongoing clinical trials testing IDO1 targeted antibodies in combination with PD-1/PD-L1 checkpoint inhibitors.

Clinical Trial	Cancer Type	Intervention	Phase	IDO1Agent	Anti-PD-1/PD-L1	Primary Outcomes	N. Patients
NCT03322540	Metastatic NSCLC	epacadostat + pembrolizumab, epacadostat + placebo	II	epacadostat	pembrolizumab	ORR	154
NCT03322566	Metastatic NSCLC	epacadostat + pembrolizumab, +/- chemotherapy	II	epacadostat	pembrolizumab	ORR	23
NCT03347123	Advanced or Metastatic solid tumors	epacadostat + nivolumab, + ipilimumab /lirilumab	I/II	epacadostat	nivolumab	TEAE, ORR	11
NCT05077709	Metastatic NSCLC	IO102-IO103 + pembrolizumab	II	IO102-IO103	pembrolizumab	ORR, PFS	90
NCT03343613	Solid tumors	LY3381916 + LY3300054	I	LY3381916	LY3300054	DLT	60
NCT03562871	NSCLC	IO102 + pembrolizumab	I/II	IO102	pembrolizumab	DLT, ORR	108

## Combination with oncolytic virus

### Oncolytic virus and TME

Oncolytic viruses (OV) are typically engineered from existing virus models and repurposed to target cancer cells. A promising therapeutic PD-1/PD-L1 combination approach involves the use of OV. To promote immunogenic cell death (ICD), the virus first must replicate exclusively in cancer cells and then promote antitumor responses *via* activation of mDC and T cells. Secretion of damage-associated molecular patterns such as adenosine triphosphate (ATP) and high-mobility group box protein 1 (HMGB1) are usually a characteristics of such responses (64, 65). Many virus models have been explored as potential therapies. Some of the more studied models include measles virus, retroviruses, herpes simplex viruses, adenovirus, bovine papillomavirus, among others. As stated, a major limitation of PD-1/PD-L1 inhibitors is the lack of patients responding to treatment. To effectively increase the rate of successful treatment outcomes, better understanding of the TME is needed. Benefits of ICI appear most successful with tumors categorized by high PD-L1 expression, increased mutational burden, and high level of TILs. These tumor types are classified as treatment-receptive tumors otherwise known as immunologically “hot” (66, 67). On the other hand, tumors classified as immunologically “cold” tumors demonstrate reduced therapeutic benefit. These tumors, express low or no TSAs, have decreased TILs density, and a low rate of suppressive immune-cell subtypes infiltrating deep tumor regions including myeloid-derived suppressor cells, regulatory T cells, NK cells, neutrophils, or macrophages. Additionally, they demonstrate low expression of immune-suppressive molecules such as (including IL-10, IDO, CD73, PD-L1, and prostaglandin E2) (68–72). A promising approach for combination ICB would ideally alter these “cold” tumors into “hot” tumors. OV therapy can heat up immunologically “cold” tumors by enabling ICB and converting immunosuppressive cells to a pro-inflammatory phenotype to effectively break the immune tolerance of the TME (73). Recently, breakthroughs and discoveries have allowed for better understanding of the mechanism behind OV therapy. It is now understood that the clinical efficacy of OVs is highly dependent on the vaccine’s ability to transform tumors into biological “vaccine factories”. OVs can promote the recruitment and activation of lymphocytes, upregulate the expression of PD-1/PD-L1 effectively increasing efficacy and downregulating resistance of ICI, as well as alter components of the antitumor immune response including small, e.g., uric acid) (74), ATP, protein mediators such as HMGB1 and IFN signaling (75, 76). The mechanisms inducing these benefits involve tumor lysis, TME alteration, TIL activation, and recruitment, triggering of immune responses mediated by innate immune cells and CB8+ T cells through antigen binding, as well as inhibition of tumor angiogenesis, neovascularization, and other such vascular modifications (77, 78). Through these responses, OV treatment can lead to increased neoantigen spreading and tumor antigen presentation, infiltration of NK cells and T cells into the TME, and increase T-cell effector function leading to a phenomenon known as the “bystander effect” both at proximal and distal sites of tumors (79). The results from one study of intra-tumoral mJX-594 treatment targeting granulocyte-macrophage colony-stimulating factor (GM-CSF) gene showed a highly altered TME, while promoting suppressed growth of tumors treated with ICB (80). These promising results led to an increase response to immunotherapy treatment. Furthermore, the combination of anti-PD-1 mAb and mJX-594 reduced tumor growth by 70%, whereas anti-PD-1 and mJX-594 monotherapy delayed tumor growth by 23% and 44%. The combination of TME alteration with PD-1 therapy is a promising approach to increasing PD-L1 expression and tumor-associated antigens, therefore, improving efficacy and the percentage of patients affected by anti-PD-1 inhibitors alone.

### Coxsackievirus A21

Coxsackievirus A21 is another OV targeting ICAM-1 on tumor cells. A phase Ib KEYNOTE-200 trial of coxsackievirus A21 in combination with Pembrolizumab demonstrated good tolerability with no dose-limiting toxicities and no grade 4/5 AEs (81). Currently, the available data show medium OS of 9.5 months, ORR of 23%, and 33% for patients with ALK-negative and EGFR-negative NSCLC. The final results of the study are awaited. Notable increases in PD-L1 tumor levels were observed indicating combination OV with ICI could have additive effects. Current findings indicate that a combination of anit-PD-1 mAb with an optimal dose of OV does not significantly increase toxicity and, in most cases, is tolerated with grade ≥3 AEs. However, a major setback is acquired resistance that arises after multiple therapies, and, generally, therapy becomes ineffective after the third dose of treatment. Nonetheless, the ongoing clinical trials testing OV in combination with ICB in NSCLC are highly anticipated and summarized in **Table 5**.

**Table 5:** Ongoing clinical trials testing oncolytic virus in combination with PD-1/PD-L1 checkpoint inhibitors.

Clinical Trial	Cancer Type	Intervention	Phase	Oncolytic Virus Vector	Anti-PD-1/PD-L1	Primary Outcomes	N. Patients
NCT02879760	NSCLC	Ad-MAGEA3 injection + MG1-MAGEA3 infusion + pembrolizumab infusion	I/II	MG1-MAGEA3	pembrolizumab	AE, ORR	16
NCT03004183	Metastatic NSCLC	ADV/HSV-tk + valacyclovir + radiation + pembrolizumab	II	ADV/HSV-tk	pembrolizumab	ORR	57
NCT02824965	Advanced NSCLC	CVA21 + pembrolizumab	I	CVA21	pembrolizumab	TEAE	11
NCT03767348	NSCLC	RP1 + nivolumab	II	RP1	nivolumab	AE, SAE, DLT, ORR, MTD	300
NCT04725331	NSCLC	BT-001 + pembrolizumab	I/II	BT-001	pembrolizumab	AE, RDPB, ORR, DCR	48
NCT04355806	NSCLC	Inactivated trivalent influenza vaccine + nivoluamb /pembrozliumab /atezolizumab, durvalumab	I	Inactivated trivalent influenza vaccine	Inactivated trivalent influenza vaccine	ADCC, irAE, PFS, OS	160

## Combination with immune modulators

### CTLA4

Cytotoxic T-lymphocyte–associated protein 4 (CTLA-4) a member of the immunoglobulin superfamily and encodes a protein, which transmits an inhibitory signal to T cells (82). Ipilimumab is an immune-checkpoint inhibitor targeting CTLA-4, which is first approved for use as monotherapy in metastatic melanoma (83). Previous research revealed that the combination of ipilimumab with nivolumab has demonstrated superior efficacy compared with nivolumab alone in patients with advanced melanoma (84, 85). Similarly, phase 1 and 2 studies in patients with untreated advanced NSCLC showed promising early results with nivolumab plus ipilimumab, and recent phase 3 trials in this population demonstrated superiority of the combination either alone or with chemotherapy compared with chemotherapy alone (86–89). Nivolumab plus ipilimumab combined with chemotherapy (2 cycles) have been approved as first-line treatment for patients with metastatic or recurrent NSCLC, with no EGFR, or ALK genomic tumor aberrations in many countries (90). However, in this phase 3 randomized clinical trial, ipilimumab plus nivolumab did not improve outcomes in patients with advanced, pretreated, immune-checkpoint inhibitor-naive lung squamous cancer (91). Several studies suggested ipilimumab plus pembrolizumab do not improve efficacy and are associated with greater toxicity as first-line and second-line or later therapy treatment for NSCLC, suggesting ipilimumab plus pembrolizumab may not be a well choice (92, 93). In a phase 1b trial, preliminary efficacy of atezolizumab, a PD-L1 inhibitor, plus ipilimumab were observed in metastatic NSCLC, and the combination had manageable toxicity, with a safety profile consistent with those of the individual agents (94). Overall, anti-PD-1/PD-L1 therapy plus anti-CTLA-4 therapy could be promising for advanced NSCLC, but the proper combination strategy is still needed to be explored. The ongoing clinical trials testing the combination of anti-PD-1/PD-L1 and anti-CTLA-4 therapy in NSCLC are summarized in **Table 6**.

**Table 6:** Ongoing clinical trials testing anti-CTLA-4 inhibitors in combination with PD-1/PD-L1 checkpoint inhibitors.

Clinical Trial	Cancer Type	Intervention	Phase	CTLA-4 Agent	Anti-PD-1/PD-L1	Primary Outcomes	N. Patients
NCT04140526	NSCLC, Solid Tumors	ONC-392, +/- pembrolizumab	IA/IB/II	ONC-392	pembrolizumab	DLT, MTD, AE, RP2D	468
NCT04043195	NSCLC	nivolumab + oxaliplatin + ipilimumab	I/II	ipilimumab	nivolumab	ORR	30
NCT05187338	NSCLC, Solid Tumors	ipilimumab + pembrolizumab + durvalumab	I/II	ipilimumab	pembrolizumab + durvalumab	AE, PFS, DCR, DOR	100
NCT04606472	NSCLC, Solid Tumors	SI-B003	I	SI-B003	SI-B003	DLT, MTD, MAD, AE, RP2D	159
NCT03377023	NSCLC	ipilimumab + nivolumab +nintedanib	I/II	iipilimumab	nivolumab	MTD, ORR	68

### LAG-3

Lymphocyte activation gene-3 (LAG-3) is a cell surface receptor expressed on activated NK cells, B cells, and T cells. Its high binding affinity to the MHC-II triggers important T-cell functions including T-cell proliferation, activation, cytolytic activity, cytokine production, and other functions (95). In cancer therapy binding between LAG-3/MCH-II triggers anti-immune response including tumor escape, decreased production of cytokines, and a reduction in CD8+ T cells response (96). However, the exact mechanism at which this immune escape occurs is unclear. Overexpression of LAG-3 has been associated with exhaustion of the immune system and is emerging as a new checkpoint inhibitor target. Furthermore, targeting LAG-3 alongside PD-1 has been shown to strengthen immune response (97). A recent study tested 20 NSCLC tumor tissue samples for identification of potential biomarkers. They found that TILs in the tumor region had increased levels of both LAG-3 and PD-1 suggesting the apparent synergy and benefit of dual checkpoint blockade therapy in NSCLC (98). In a phase I/II trial testing LAG525, an anti-LAG-3 agent, in combination with PDR001, an experimental anti-PD-1 agent showed conversion of immune cold to immune-activated TME and durable response in 12 patients (11 partial response and one complete response). It was noted that LAG525 alone and LAG525 in combination with PDR001 demonstrated high progressive disease of 79% and 67%, respectively (99). A follow up study was recently completed (NCT02460224). However, statistic data were not fully disclosed. In a phase I/II testing relatlimab, an experimental anti-LAG-3 agent in combination with nivolumab showed ORR 11.5% and disease control rate (DCR) of 49% with acceptable AEs (grade ≥ 3 in 10% of patients) (100). Additionally, FDA approval of Opdualag for melanoma, a fixed-dose combination of relatlimab and nivolumab demonstrated increased PFS of 10.1 months for Opdualag compared to 4.6 months for the nivolumab monotherapy (NCT03470922). Although not approved for NSCLC, this first ever phase III trial of a LAG-3 antibodies demonstrates the validity of the methodology behind treatment. Promising combination approaches for NSCLC are now being tested in several ongoing clinical trials including phase I/II studies of neoadjuvant nivolumab in combination with relatlimab, eftilagimod alpha in combination with pembrolizumab (NCT03625323) (101), BMS-986,016 in combination with nivolumab (NCT01968109), and more which are summarized in **Table 7**.

**Table 7:** Ongoing clinical trials testing anti-LAG-3 inhibitors in combination with PD-1/PD-L1 checkpoint inhibitors.

Clinical Trial	Cancer Type	Intervention	Phase	LAG-3 Agent	Anti-PD-1/PD-L1	Primary Outcomes	N. Patients
NCT03625323	NSCLCHNSCC	eftilagimod alpha + pembrolizumab	II	eftilagimod alpha	pembrolizumab	ORR	189
NCT01968109	NSCLC	BMS-986213, +/- nivolumab	I/IIa	BMS-986016	nivolumab	AE, SAE, ORR, DCR, DOR	1499
NCT02460224	Advanced Solid Tumors	LAG525, +/- PDR001	I/II	LAG525	PDR001	DLT, ORR	490
NCT04140500	NSCLC, Solid Tumors	RO7247669	I	RO7247669	RO7247669	DLT, AE, ORR, DOR, PFS	320
NCT03849469	NSCLC	XmAb^®^22841, + pembrolizumab	I	XmAb^®^22841	pembrolizumab	AE	242

### OX-40

OX-40 is a type 1 transmembrane glycoprotein found on the surface of activated CD8+ and CD4+ T cell and part of the tumor necrosis factor receptor family. Activation of the OX-40/OX-40L axis acts as a costimulatory signal triggering T cell survival and division for both effector and memory cell populations against target antigens (102). Additionally, OX-40 activation suppresses proliferation and functionality of Tregs, preventing the TGF-β–mediated conversion of CD4+ T cells into Tregs, further increasing immune activity (103). Therefore, activation of OX-40 has been sought out as a therapeutic target for cancer immunotherapy. OX-40 inhibitors have shown to be effective in immunogenic tumors on some cancer cells lines including MCA303 (sarcoma tumors), SM1 (breast cancer), and CT26 (colon carcinoma tumors) (104). However, in immunogenic cold tumors, data were less promising. Considering the variability of immunogenicity in tumors from patient to patient and cancer type to cancer type (105), enhancing OX-40 efficacy *via* combination approaches has been explored. Some studies have demonstrated that the combination of anti-PD-1 ICI in combination with OX-40 inhibitors is feasible. Evidence from an OX-40 antagonist trial demonstrated synergic effects on different types of murine models with sequential combination of anti-PD-1 ICI showing 40% survival rate at day 100, compared to 0% survival rate with no treatment (106). A recent biomarker analysis study with 139 NSCLC patients showed that high PD-1 expression is negatively correlated with TILs OX-40 and OX-40L expression (0.250 and 0.386), according to linear regression models (107). This indicates that some patients with low PD-1 expression may have higher OX-40 and OX-40L expressions, suggesting OX-40 inhibitors have potential to be effective in patients at lower chances of benefiting from anti-PD-1 inhibitors monotherapy. In a phase I trial of anti-OX-40 mAb, GSK3174998 administered as monotherapy or combined with pembrolizumab demonstrated no dose-limiting toxicities, indicating feasibility in solid and NSCLC cancer models (108). A phase I trial results testing MEDI0562, a humanized IgG1k OX-40 mAb in combination with durvalumab or tremelimumab, an anti-CLTA-4 inhibitor, for patients with late-stage solid tumors demonstrated OS of 17.4 and 11.9 months, respectively (109). A different triple combination approach testing BMS-986178, an OX-40 agonist, with ipilimumab with/without nivolumab in phase I/II demonstrated grade 3–4 AEs in six out 79 patients (8%) in anti-OX-40 with nivolumab. However, no clear efficacy benefit was observed when compared to nivolumab monotherapy (110). Further ongoing clinical trials are awaited to get a better understanding of the mechanism behind this novel combination approach and are summarized in **Table 8**.

**Table 8:** Ongoing clinical trials testing anti-OX-40 inhibitors in combination with PD-1/PD-L1 checkpoint inhibitors.

Clinical Trial	Cancer Type	Intervention	Phase	OX40 Agent	Anti-PD-1/PD-L1	Primary Outcomes	N. Patients
NCT02528357	Neoplasms	GSK3174998, +/- pembrolizumab	I	GSK3174998	pembrolizumab	SAE, DLT,	141
NCT02410512	Neoplasms	MOXR0916 + atezolizumab	I	MOXR0916	atezolizumab	DLT, AE	610
NCT02554812	Advanced Cancer	avelumab, + PF-04518600, +/- utomilumab	II	avelumab	utomilumab	DLT, ORR	398
NCT02221960	Recurrent or Metastatic Solid Tumors	MEDI6383, +/- durvalumab	I	MEDI6383	durvalumab	AE, SAE	39
NCT03241173	Advanced or metastatic NSCLC	INCAGN01949 + nivolumab	I/II	INCAGN01949	nivolumab	TEAE, ORR	52
NCT04198766	Advanced or Metastatic NSCLC	INBRX-106, +/- pembrolizumab	I	INBRX-106	pembrolizumab	AE, SAE, MTD, RP2D	150
NCT03758001	Advanced Solid Tumors	IBI101, +/- sintilimab	I	IBI101	sintilimab	AE	38
NCT04215978	Advanced Soldi Tumors	BGB-A445, +/- tislelizumab	I	BGB-A445	tislelizumab	AE, SAE, MTD, RP2D, ORR	68
NCT02705482	Advanced Solid Tumors	MEDI0562	I	MEDI0562	durvalumab	AE	58
NCT02737475	Advanced Cancer	BMS-986178, +/- nivolumab, +/ipilimumab	I/II	BMS-986178	nivolumab	AE, SAE,	171

### TIGIT

A target for immunotherapy in NK-cells that has been grabbing a lot of attention is the poliovirus receptor (CD155). The binding of this protein in the body can lead to either improve immune response or increased immune suppression in cancer patients (111, 112). CD155 acts as a ligand and binds three ways in cancer patients triggering three distinct responses (113, 114). One of its binding domains is a glycoprotein called DNAM-1. DNAM-1 is commonly expressed on NK and CD8+ T cells and binding with CD155 leads to an increased anti-tumor response by activation of immune cells. The second binding domain is the TIGIT immunoreceptor for which CD155 has a higher affinity than DNAM-1. However, binding leads to an immunosuppressive response. TIGIT is quickly becoming a new immune checkpoint target in combination with PD-1/PD-L1. One study showed that high TIGIT/DNAM-1 ratio in Treg cells found in tumor tissue demonstrated a correlation to poor clinical outcomes after treatment with anti–PD-1 ICB (115). Furthermore, combination of anti-TIGIT with other therapies, mainly PD-1/PD-L1 ICB, have shown to overcome the limited efficacy of anti-TIGIT mAb alone in subcutaneous mouse tumors (116, 117). In a randomized phase II study of advanced NSCLC patients treated with tiragolumab, an anti-TIGIT antibody, combined with atezolizumab results showed that compared to a control group there was significant increase in PFS (5.6 *vs*. 3.9 months) and ORR (37.3% *vs*. 20.6%) and after 10.9 months follow up (118). Following these results, the FDA granted approval of tiragolumab in combination with atezolizumab for first line treatment in metastatic NSCLC categorized with high PD-L1 expression. Currently, ongoing clinical trials are testing anti-TIGIT mAb in combination with PD-1/PD-L1 therapy, including locally advanced or metastatic NSCLC (NCT03563716), advanced or metastatic solid cancers (NCT02913313), untreated locally advanced unresectable or metastatic NSCLC (NCT04294810), and more with a full list summarized in **Table 9**. The third type of binding occurs with CD96, and while less data are available regarding its interaction with CD155 in humans, mice models suggest that the binding promotes tumor escape from the immune system (119, 120). These mechanisms, however, are more complex and have more than one ligand/receptor combination. Recently, TIGIT and DNAM-1 have been found to bind to a ligand called CD112, which also binds to the immune-cell receptor CD112R (PVRIG) causing DNAM-1 and TIGIT to compete for the binding of CD112 (121–123). The DNAM-1/TIGIT/CD96 pathways offer new ways to improve immune-cell anti-tumor response. However, their mechanism in cancer therapy is not fully understood yet.

**Table 9:** Ongoing clinical trials testing anti-TIGIT inhibitors in combination with PD-1/PD-L1 checkpoint inhibitors.

Clinical Trial	Cancer Type	Intervention	Phase	Anti-TIGIT Agent	Anti-PD-1/PD-L1	Primary Outcomes	N. Patients
NCT05014815	NSCLC	ociperlimab, +/- tislelizumab, + chemotherapy/placebo	II	ociperlimab	tislelizumab	PFS	200
NCT04294810	NSCLC	tiragolumab, + atezolizumab/placebo	III	tiragolumab	atezolizumab	PFS, OS	635
NCT03563716	NSCLC	tiragolumab, + atezolizumab/placebo	II	tiragolumab	atezolizumab	ORR, PFS	135
NCT04513925	NSCLC	tiragolumab, +/- atezolizumab	III	tiragolumab	atezolizumab	PFS,	800
NCT03628677	Solid Tumors	domvanalimab, +/- zimberelimab	I	domvanalimab	zimberelimab	TEAE	74
NCT04262856	NSCLC	zimberelimab, +/- domvanalimab, +/- etrumadenant	II	domvanalimab	zimberelimab	ORR, PFS	150
NCT02964013	Neoplasms	vibostolimab, +/pembrolizumab, +/- chemotherapy	I	vibostolimab	pembrolizumab	DLT, AE	492
NCT04165070	NSCLC	vibostolimab, +/- pembrolizumab, +/- chemotherapy	II	vibostolimab	pembrolizumab	ORR	270
NCT02913313	Solid Tumor	BMS-986207, +/- nivolumab, +/- ipilimumab	I/II	BMS-986207	nivolumab	AE, SAE, ORR, mDOR, PFS	130
NCT03260322	Solid Tumors	ASP8374, +/- pembrolizumab	I	ASP8374	pembrolizumab	DLT, AE, irAE	169
NCT03119428	Advanced or Metastatic Solid Tumors	OMP-313M32, +/- nivolumab	I	OMP-313M32	nivolumab	DLT, AE	33

### IL-2

IL-2 is a cytokine that plays multiple roles in the activation and stimulation of the immune system. Binding occurs between the IL-2 ligand and IL-2 receptor (IL-2R) which is widely expressed on the surface of many immune cells types, including Foxp3+ regulatory T cells, CD4+ and CD8+ T cells, B cells, and NK cells (124). Upon activation, CD8+ and CD4+ T cells secrete large amounts of IL-2 in autocrine and paracrine pathways to recruit neighboring IL-2R+ cells. These circulating IL-2 molecules also bind to interleukin 2 receptor α-chain (IL-2Rα; CD25) expressed on Treg cells, which restrain immune responses to self and foreign antigens. There exist varying binding affinities of the IL-2/IL-2R pathway. IL-2R is composed of different subunits, IL-2Rα, IL-2Rβ (CD122), and IL-2Rγ (CD132), which are found at varying concentrations on the surface of different species of immune cells (125–127). In the body, low concentrations of IL-2 bind to the high-affinity receptors found on Treg cells leading to an immunosuppressive response. Only in higher concentrations can IL-2 bind to the lower affinity IL-2R found on CD8+ T-cells and NK-cells. These mechanisms are believed to help regulate immune response and prevent T-cell overstimulation from IL-2 signals and consistent TCR stimulation from tumor and self-antigens, which have shown to lead to T cell exhaustion or Fas (CD95)-mediated apoptosis (5). To overcome these regulatory mechanisms in cancer therapy, one approach is utilizing a combination strategy with PD-1/PD-L1 ICI. A study using TCB2, a newly engineered IL-2 antibody to specifically target the receptor of T cells and NK cells has been tested on mice models. Noticeably, it was shown that when a suboptimal dosage of anti-PD-1 mAb was used in combination with hIL-2/TCB2c, all seven mice models were tumor free after 19 days of treatment (128). A single-arm, phase I-dose escalation trial testing nivolumab combined with NKTR-214, a CD122-preferential IL2 pathway agonist, demonstrated ORR at 59.5% (22/37) and complete response at 18.9% (seven out of 37) for solid tumors patients including NSCLC. Additionally, analysis of tumor biopsies on a genetic and cellular level showed increased numbers of activated CD8+ T cells, without Treg cell activation (129). An ongoing dose-expansion phase II trial with optimal doses of 0.006 mg/kg and 360 mg every 3 weeks of NKTR-214 with nivolumab, respectively, demonstrated acceptable toxicity, while also promoting treatment efficacy regardless of PD-L1 levels (NCT02983045). It was reported that eight patients (21%) had grade 3/4 AEs with no cases of deaths from treatment. Total ORR for the various tumor types (melanoma, renal cell carcinoma, and NCSLC) and dose cohorts were 59.5% (22 out of 37), with complete response in seven patients (18.9%). Gene and cellular expression analysis of tumor samples demonstrated increased cytotoxicity, activation, and infiltration of CD8+ T cells without triggering Treg cell activation. Another ongoing clinical trial including NKT-214 with pembrolizumab in solid tumors (NCT03138889), IL-2 in combination with nivolumab for advanced NSCLC (NCT03215810). On a final note, high IL-2 circulating levels have been correlated with improved OS and response to PD-1/PD-L1 blockade in NSCLC demonstrating a potential for improved treatment outcomes in patient groups with higher risk of poor prognosis to anti-PD-1/PD-L1 inhibitors (130).

## Combination adoptive cell transfer

### CAR T cell

In recent years, T cell therapy has been gaining momentum as a new immunotherapy approach. Perhaps, the most popular model, chimeric antigen receptors (CAR) T cell treatment utilizes the patient’s own T cells and genetically modifies them to target cancer cells. Briefly, CAR were expressed on the patient’s T cells surface typically using an unarmed virus. Finally, they are injected back into the patient with hopes of giving a lasting antitumor response. In 2017, CD19 CAR T cell became the first FDA approve adoptive T cell transfer therapy after remarkable therapeutic effects in large B cell lymphoma or acute lymphoblastic leukemia (131, 132). Recently, reports have shown that the potential of CAR T cell immunotherapy in NSCLC (133). For NSCLC the most common TSA targets include EGFR, mesothelin, MUC1, CD80/CD86, PD-L1, inactive tyrosine-protein kinase transmembrane receptor (ROR1), carcinoembryonic antigen (CEA), among others (134). One CAR T cell study targeting EGFR in advanced NSCLC (NCT01869166) had reported preliminary results showing 45.5% (five out of 11) of patients achieved stable disease, and 18.2% (2/11) achieved partial response (135). CAR T cells targeting these antigens have been promising. However, some key challenges relating to CAR T cells include manufacturing concerns, restricted trafficking, infiltration and activation within tumors, severe toxicities, insufficient persistence *in vivo*, heterogeneity, and antigen escape (136).. In efforts to increase efficacy, some studies have attempted combining anti-PD-1 inhibitors with CAR T cell therapy. In a study testing CAR T cell combined with anti-PD-1 mAb, results showed significantly increased growth and survival inhibition of two different HER2+ transgenic mouse tumor models when compared to either treatment in monotherapy (137). A study for anti-MUC1 CAR T cells in combination with engineered PD-1 deficient T cells in NSCLC patients demonstrated 33% (two out of six) patients had significant shrunken tumors after 4 weeks of treatment (138). A clinical trial testing PD-1 knockout (KO) CAR T cells in NSCLC proved to be safe and well-tolerated by all patients, demonstrating stable disease in 55% (11 out of 20) patients (139). It is important to note that the combination of PD-1/PD-L1 targeted pathways and the CAR T cell treatment in clinical trials are still in early development, and there is a lack of necessary data to draw valid conclusions (140). Nonetheless, new promising methodologies are being awaited with CAR T cell therapy including CRISPER CAS-9 PD-1-knockout–modified CAR T cells and engineered CAR T cells with the capabilities of secreting PD-1-blocking single-chain variable fragments (scFv) (141, 142). With new innovations in genetic engineering, CAR T cell therapy may utilize PD-1/PD-L1 ICI or genetic alterations to improve efficacy in combination treatment.

### NK cell

NK cells are another rising form of immunotherapy found in combination with PD-1– based inhibitor drugs. They are natural cytotoxic cells found in the body and require no stimulation to activate. They work by binding to receptors or antibodies present on abnormal cells such as cancer cells or TME. Cancer therapy has explored the use of autologous or allogeneic NK cell-based therapy, transplanting different subsets of NK cell populations into a cancer patient to achieve an anti-tumor response. Moreover, some studies demonstrate the benefit of combining NK cell therapy with ICI. One study suggests that PD-L1 mAb can directly upregulate and activate the cytotoxic effector functions of NK cells without any correlation to PD-L1 tumor status (143). Furthermore, *via* the AKT signaling pathway, PD-L1 mAbs were found to enhance NK cell function and prevent cell exhaustion, through upregulation of PD-L1 expression on NK cell surface. Recent study demonstrated that the combination of mHsp-70 targeting autologous NK cells therapy with nivolumab and radiochemotherapy was well tolerated with tumor progression or metastases not detectable 33 months post-diagnosis for one NSCLC patient (144). A study testing pembrolizumab in combination with allogeneic NK cells in comparison with pembrolizumab monotherapy in advanced NSCLC results demonstrated higher PFS (6.5 months *vs*. 4.3 months) and higher median OS (5.5 months *vs*. 13.3 months) (145). Several courses of NK cell injection demonstrated better OS (18.5 months) compared to single-course infusion (13.5 months), notably with the combination approach having much higher median OS and PFS in PD-L1 tumor portion score (TPS) ≥50%. One purpose mechanism for this increased efficacy involves the mechanism at which expanded NK cells engage with the TME. Through a contact-independent mechanism, NK cells promote endogenous TILs and upregulation of PD-L1 TPS (146). The difference between expanded NK cells and NK cells, which found naturally in the body, is that they are less susceptible to tumor suppression, therefore allowing them to upregulate immune response through binding with PD-L1 tumors. NK cells therapy can potentially turn nonresponding tumors into more susceptible to PD-1/PD-L1 treatment. Currently, an ongoing phase I clinical trial is testing the combination of PD-1/PD-L1, chemotherapy, and FT538 allogeneic NK cell therapy in advanced solid cancer (NCT05069935). These studies demonstrate a correlation between NK cell function and PD-1/PD-L1 inhibition; however, there lacks a mechanistic *in vivo* studies explaining these responses (147). Another issue with the use of autologous NK cell transfer is that the procedure is often expensive as each treatment is personalized and based on cells from the patient or donor. Lastly, the personalized process is time-consuming and due to the nature of the disease might prove inapplicable in some cases. NKG2A is an inhibitory signaling receptor found on the surface of NK cells. Similar to the NKG2D ligand, efforts have been focused on increasing the efficacy of NK cell transfer treatment by targeting this axis. A popular anti-NKG2A antibody, known as monalizumab, is now being tested with anti-PD-1 blockade. One clinical trial testing these agents demonstrated the feasibility of combination treatment with ORR of 8% and a DCR at 16 weeks of 31% (148). *In vitro* and *in vivo* blocking on mice models demonstrated that when NKG2A and PD-1 blockers were combined, results showed a significant increase in the rate of tumor regression and anti-tumor immunity (149, 150). The NKG2A/NKG2D axis offers a viable way to increase the efficacy of PD-1 treatment by improving NK cell function and homeostasis in the body. In addition, combination immunotherapy with anti-PD-1/anti-PD-L1 and anti-NKG2 to improve efficacy of previously less effective anti-PD-1 treatments characterized by high levels of circulating sMIC should be explored.

### Modified NK cell

To get around these issues, new forms of genetically engineered NK cells are now being brought into the market and sold as “off-the-shelf” universal treatment. A common type uses CAR NK cells, which are engineered to present antibodies on their surface and bind to antigens on the surface of cancer cells. CAR NK cell therapy does not require matching HLA donors unlike allogeneic NK cells or CAR T cell transplants and has a higher safety profile in this sense. This allows for large-scale commercial treatments to be manufactured. NK cells prove more challenging to extract and isolate than T cells. However, a line of NK-92 cells has been found to expand easily *in vitro* and engineered to present CAR on their surface. In theory, this CAR-NK cell line would be comparable to a universal CAR T cell treatment but, simply adding CAR receptors to NK cells is proving to be insufficient. Most CAR models are engineered based on T-cell structure and functionality. For example, CAR models containing co-stimulatory CD28 domains intended for T cells are commonly used in NK cells despite having no effect or activating NK cells (151). Currently, the latest advances in NK cell therapy are working to increase the efficacy of treatment through a better understanding of the NK cell activation mechanism and newly modified NK cell treatments. One clinical trial underway is testing the use of iPSC-derived NK cells combined with pembrolizumab, nivolumab, and atezolizumab in late-stage solid tumors, including NSCLC (NCT03841110). Another approach, derived from several studies, utilizes the NKG2D ligand, which plays a critical role in NK cell activation. The most common molecules released from the NKG2D ligand family in tumors are MICA and MICB, otherwise, referred to as sMIC. In a study targeting tumor-derived soluble NKG2D MIC molecules, simultaneously targeting sMIC with a PD-1/PD-L1 inhibitor resulted in enhanced infiltration, intrinsic function, and proliferation of CD8+ T cells with a TILs score recorded at 32.5% compared to 21% TILs of anti-PD-L1 monotherapy (152).. This study suggests through these antibodies targeting sMIC can increase the efficacy of T cell therapy in combination with ICB. Furthermore, the NKG2D ligand is either not induced or induced in low levels under normal conditions and that it is overexpressed only on the oncogenic cell surface or in the TME (153–159). This allows specific ligand to act not only as a target receptor for elimination of cancer cells but also as an indicator of such abnormalities [133]. However, this approach is not fully proven. Elevated levels of circulating NKG2D are correlated with poor outcomes in anti-PD-1/PD-L1 clinical trials (160). This is, in part, due to a process of proteolytic shedding (161–164), in which the tumor releases the ligands from its cell surface leading to powerful immune-suppressive responses and disturbing NK cells homeostatic maintenance and function (165, 166). A new strategy for the creation of universal super NK cell therapy has been proposed. This new strategy involves the use of aptamer-equipped NK cells engineered *via* metabolic glycan biosynthesis and, thus, avoids the use of genetic alteration (167). This approach allows for specific tumor targeting therapies *via* modified receptors on the NK cell surface. Furthermore, to enhance immunotherapy, PD-L1 targeting aptamers were also modified on the NK cell surface to regulate PD-1/PD-L1 signaling. This lead substantial upregulation of PD-L1 expression in HepG2 cells improving ICB. Although the mechanism remains unclear, it is known that increased levels of PD-L1 expression have been correlated with higher success from immunotherapies (168). Additionally, imaging of intravital tumor sites showed high levels of modified NK cells in deep tumor regions, indicating better infiltration and therapeutic efficacy for solid tumors. This approach of increasing infiltration in solid tumors *via* chemical engineering and has several benefits. First, the engineering strategy is simple and efficient and does not require genetic modification that reduces the risk for side effects in clinical applications. Secondly, the NK cells are biodegradable biocompatible, and no signs of toxicity was detected on mice models in the study. Third, the strategy is universal and by simply changing the target aptamer, different types of solid tumors can be targeted. This is made possible by systematically increasing ligands through exponential enrichment technology, while screening for aptamers from various cancer cell types giving a dataset of aptamers/cancer compatible combinations (169–173). Fourth and perhaps the most remarkable benefit, the study found aptamer-equipped NK cells to upregulate PD-L1 expression. This directly targets one of the major issues lying in targeting this axis, which was the lack of PD-L1 expression on cancer cells. For these reasons and, in particular, the upregulation of PD-L1, this new strategy of chemically modified NK cells needs further testing and to test the feasibility of combination with PD-1/PD-L1 inhibitors. Aptamer-equipped glycan biosynthesis NK cells may potentially be the first step in providing successful universal NK cell transplants. CAR NK cells and iPSC NK cells also provide a pathway to universal treatment, however, face issues with functionally. Additionally, autologous NK cells have shown promising clinical trial data when combined with pembrolizumab and nivolumab. These promising results, notably, are preliminary mechanistic studies that show potential for NK cells in combination with PD-1/PD-L1 inhibitors. More clinical trial data are highly anticipated with these ongoing combination approaches summarized in **Table 10**.

**Table 10:** Ongoing clinical trials testing cell-based therapies in combination with PD-1/PD-L1 checkpoint inhibitors.

Clinical Trial	Cancer Type	Intervention	Phase	Cell Therapy	Anti-PD-1/PD-L1	Primary Outcomes	N. Patients
NCT03525782	NSCLC	CAR-T Cell, +/- PD-1 knockout T-cell	I/II	CAR-T Cell, PD-1 knockout T-cell	pembrolizumab	AE, DLT	60
NCT04556669	Solid Tumor, NSCLC	Autologous aPD-L1 armored CD22-targeting CAR T cells	I	Autologous aPD-L1 armored CD22-targeting CAR T cells	Autologous aPD-L1	irAE	30
NCT05069935	Solid Tumor	FT538, +/- avelumab, +/- atezolizumab, +/- nivolumab, +/-pembrolizumab	I	FT538 allogeneic NK-Cell immunotherapy	avelumab, atezolizumab, nivolumab, pembrolizumab,	RP2D, AE	189
NCT03841110	NSCLC, Advanced Solid Tumors	FT500, +/- nivolumab, +/- pembrolizumab, +/- atezolizumab, +/- chemotherapy	I	FT500, an allogeneic, iPSC-derived Natural Killer (NK)	nivolumab, pembrolizumab, atezolizumab	DLT	37

## Probody therapeutics

Finally, a new approach to improve PD-1/PD-L1 inhibitors efficacy is using a masked peptide linker to inhibit the binding of therapeutics to normal cells, while becoming unmasked and activated in the TME exclusively. Probody therapeutics are a next generation antibody using a masked peptide to cover the fab region. Once in the TME, the fab region gets cleaved off by tumor-associated proteases exposing the PD-L1 substrate domain. One study using extracted tumor samples from cancer patients demonstrated over 90% of cancer patients had sufficient protease activity to activate treatment *in vivo* (174). Initial *in vitro* results showed that the masked Pb-TCB reduced cytotoxicity by 100,000-fold, whereas the unmasked molecule proved potent in tumor killing at proper dosing schemes (175). From the concluded preclinical and preliminary clinical studies, one Probody PD-L1 targeting compound called CX-072 has demonstrated potential to optimize cancer treatment, while minimizing toxicity (176). CX-072 is a next generation of cancer immunotherapy that offers a new way to increase the percentage of patients affected by PD-L1 targeted treatments, while potentially reducing the rate of AEs associated with PD-1/PD-L1 blockade by overlooking normal tissue (176). In a dosing-finding clinical trial (NCT03013491), CX-072 was tolerated with indications of antitumor activity in patients without high levels of PD-L1 (177). As to our current knowledge, this treatment has not been tested in NSCLC. However, it offers a new approach to increase the percentage of NSCLC patients benefiting from PD-1/PD-L1 by increasing the precision of targeted treatment consequently decreasing required minimum dose and, therefore, rates of toxic adverse effects.

## Discussion

For the past decade, ICI inhibitors and particularly PD-1/PDL-1 ICB have revolutionized NSCLC therapy. However, over 50% of patients do not respond to PD-1/PDL-1 inhibitor-based monotherapy due to low expression of PD-L1 in lung cancer patients, low numbers of TILs, and low mutational burden (178–180). Targeting specific characteristics of cancer patients alongside incorporating combination approaches based on tumor genomics and immunology data is the future of treatment. Ideally, a successful combination approach would stimulate one of the abovementioned factors associated with poor prognosis of PD-1 therapy alone. Vaccine therapy offers the potential to do just do that based on the biological mechanisms of how vaccines work. Alteration of the immune system to express certain receptors, activation of T cells against those receptors, and release of antibodies all pose the capability of producing additive antitumor response when used in combination with PD-1/PD-L1 ICI for patients who are prone to poor prognosis. However, neutralization is currently a key setback, especially with multiple doses and future emphasis should focus on exploring the immune mechanisms leading to this.

Another issue that lies with PD-1 therapies arises from NSCLC resistance to ICB. Treatment outcomes are not yet universal, but one theory shows that this might be mediated through carcinoma-associated fibroblasts (CAF), which also influence the CXCR4/CXCL12 axis (181). While the mechanism is not yet fully understood, it is hypothesized that CXCR4/CXCL12 axis and CAF may be responsible for resistance to ICB (182). For example, one ovarian mouse model with dual blockade CXCR4/CXL12 using AMD3100 and PD-1/PDL-1 showed increased effector T cell infiltration, function, and memory in tumors (183). Future advances on therapeutics targeting this axis should focus on optimal dosing and minimizing toxicity. VEGFR is another commonly overexpressed receptor found on NSCLC that can be used to overcome immune resistance to PD-1/PD-L1 drugs. A combination approach using anlotinib showed promising results in NSCLC clinical trials with significant improvements in OS and PFS. A targeted EGF TKI Erlotinib demonstrated additive effects with PD-1 drugs with a 19% ORR (48). However, a follow-up phase I/II study concluded no significant improvements in ORR between pembrolizumab monotherapy and the combination approach using Erlotinib (49). The early nature of these studies makes it difficult to conclude any solid findings. Furthermore, VEGF and EGF TKIs are prone to adaptive resistance to treatment by NSCLC, which could prove treatment in multiple doses to be ineffective or even create a more difficult disease to treat. These treatments may prove only beneficial in early-stage NSCLC since the larger the tumor gets, the harder it would be to clear it completely. If the tumor is harder to clear, more treatment dosages would be needed and, therefore, a higher likelihood for adaptive resistance to occur is present. Further data from phase I/II clinical trials found in **Table 2** are waited for better understanding of this combination approach.

A universal challenge in cancer treatment lies in tumor evasion from immune detection. ICB has the potential to down regulate immune escape mechanism as they can directly suppress or stimulate the immune system. LAG-3/MHC-II binding complex has shown to play key roles in cancer immune escape. Anti-PD-1 combined with anti-LAG-3, therefore, poses promising synergy benefits and data from various clinical trials showed promising effects from dual block therapy in NSCLC (99–101). OX-40 has been shown to downregulate Treg function, while acting as a costimulatory cytokine for CD4+ and CD8+ T cells, with both functionalities corresponding to improve immune function. This combination approach demonstrates potential to increase immune response and infiltration of T cell into tumor region, a key setback of PD-1/PD-L1 monotherapy. However, in a phase I/II of BMS-986178, an OX-40 inhibitor combined with nivolumab/ipilimumab showed no clear benefit (110). It is unclear how exactly these two therapeutic agents work together; however, due to the boost in immune response, OX-40 does have the potential to improve NSCLC treatment efficacy and should be explored further. Another approach covered used an anti-TIGIT in combination with PD-1/PD-L1. CD155 is an upcoming immunotherapy with three distinct methods of binding that could lead to three different outcomes. The highest affinity TIGIT molecule showed promising result when combined with anti-PD-L1 in a phase II study testing tiragolumab in combination with atezolizumab. IL-2 is a circulating cytokine with multiple mechanisms of action in immune response *via* the JAK-STAT, PI3K/Akt/mTOR, and MAPK/ERK pathways. Low concentration of IL-2 led to increased immune suppression by binding to Treg, while increased levels led to immune activation by binding/activating NK and CD8+ T cells and were correlated with improved OS and better response to PD-1/PD-L1 blockade in NSCLC. Therefore, this cytokine can be used in combination approach as both as a therapeutic target and biomarker. However, it also important to note that combination of immunomodulators and PD-1/PD-L1 inhibitors led to a high number of AEs in some clinical trials. Additionally, BMS-986178 and OX-40 phase I/II clinical studies showed no increased benefits. It is possible that these combination regimes might only be effective for a specific patient group, categorizes by tumor receptor expression level, TME antigen concentration and stage of disease. Nonetheless, immunomodulators combined with PD-1/PD-L1 are promising and require further review for a better understanding of the complex immune activation mechanisms.

NK cells have been gaining a lot of momentum in recent years. Many variations of the NK cell type have been tested. NK cells have showed promising results *via* autologous transplantation. However, the procedure is expensive and time-consuming. Modified CAR-NK cells conceptually seemed like a promising approach. However, CAR models designed for T cells did not activate NK cells as they would in T cells. A promising approach used a metabolic glycan biosynthesis and click reaction to chemically bind dual aptamers to target cancers and regulate PD-1/PD-L1 signaling (167). Its interchangeable aptamers pose a way to effectively target a wide variety of NSCLC mutations and regulation of the PD-1/PD-L1 axis may increase the efficacy of PD-1 inhibitor drugs. It is important to note that this therapy is in very early stages and more studies are needed to validate these findings to begin entering this combination approach into clinical trials.

Another combination approach that led to increased immune activation was OV therapy. OV therapy can be used to activate TILs, suppress tumor growth, and alter the TME. These responses pose synergy effects to patients who do not respond to PD-1/PD-L1 inhibitors well and making this combination promising. Currently, six clinical trials are ongoing of PD-1/PD-L1 combined with OV therapy, and we await the data from these trials to gain a better understanding of an optimal combination approaches. A downside of the treatment is that extensive use of OV therapy arising from repeat administrations can lead to progressively weakened response and spread of viral infection due to rapid neutralization by the immune system (184). Hosts with normal immunity have shown decreased reduced anti-tumor activity, viral clearance, and oncolytic viral replication in various pre-clinical and phase I clinical studies (185–192). OV therapy, which attempts to overcome these challenges, lies brought on by neutralization of the immune system through cell encapsulation (193), immunomodulators (194), and DNA aptamers (195, 196). The future of OV therapy needs to focus on optimal combination approaches, while minimizing neutralization and toxicity.

It is important to note that these combination strategies are the latest advances in the field of NSCLC treatment. Our current understanding of immunotherapy suggests that these treatments are promising. However, they might not be ideal for everyone. As these trials are still in early stages, results should still be treated with caution. Additionally, although not primarily focused on in this paper, chemotherapy and radiotherapy are effective cancer treatments with over 60% of patient’s diagnosis at stage III/IV receiving a dose of one or other (197). The future of ICI therapy involves understanding of tumor immunology. Tumors continuously evolve throughout the disease causing a high level of spatial and temporal heterogeneity. In addition, intra- and inter-tumor regions also express heterogeneity throughout the different stages. This heterogeneity is a key hurdle in the way of predicting treatment outcomes accurately. To mainstream cancer treatment, it now understood that analyzing tumor genomics and immunomodulating activities are critical for increasing success rates. This “new-revolution” of cancer therapy directly relies on the use of the TME and tumor immunology as predictive biomarkers for the development of optimal combination approaches. These approaches can be achieved to directly target overexpression and suppressed immune pathways. It is known that each tumor demonstrates different levels of biologic expressions, and this varies greatly from person to person. The near future of the field will orientate more towards a personalized approach to first screen tumors for key antigens in the TME and expression levels of important receptors on NSCLC, such as PD-L1 and CT-L4. Ideally, a biopsy of the tumor will also be used to perform DNA assays and determine tumor mutations. Finally, early diagnosis has proven to be a key factor in lowering the mortality rate. Awareness needs to be spread to encourage periodic testing to those higher risk groups such as those exposed to cigarette smoke. As more data will be released, it will be necessary to create a system to gather, manipulate, and utilize the findings from combination PD-1/PD-L1 in clinical trials. Using data and machine learning models to diagnosis, creating treatment combinations and dosage regimes is the future of cancer therapy and, ultimately, is a feasible method to universally treat NSCLC, while bypassing the need for a universal treatment.

## Author contributions

YW and WM designed the study and participated in coordination and project control. OM and JM wrote the manuscript. JM and OM prepared Tables. YW and WM revised the manuscript. All authors contributed to the article and approved the submitted version.

## Funding

This work was supported by National Cancer Institute (1R01CA230339) subaward and 1R37CA255948), Natural Science Foundation of Jiangsu Province (BK20210068), and the High-end Medical Expert Team of the 2019 Taihu Talent Plan (2019-THRCTD-1).

## Conflict of interest

The authors declare that the research was conducted in the absence of any commercial or financial relationships that could be construed as a potential conflict of interest.

## Publisher’s note

All claims expressed in this article are solely those of the authors and do not necessarily represent those of their affiliated organizations, or those of the publisher, the editors and the reviewers. Any product that may be evaluated in this article, or claim that may be made by its manufacturer, is not guaranteed or endorsed by the publisher.
